# Species Diversity Distribution Patterns of Chinese Endemic Seed Plants Based on Geographical Regions

**DOI:** 10.1371/journal.pone.0170276

**Published:** 2017-01-23

**Authors:** Jihong Huang, Keping Ma, Jianhua Huang

**Affiliations:** 1 Key Laboratory of Forest Ecology and Environment, the State Forestry Administration, Institute of Forest Ecology, Environment and Protection, Chinese Academy of Forestry, Beijing, China; 2 Co-Innovation Center for Sustainable Forestry in Southern China, Nanjing Forestry University, Nanjing, China; 3 State Key Laboratory of Vegetation and Environmental Change, Institute of Botany, Chinese Academy of Science, Beijing, China; 4 School of Economics, Minzu University of China, Beijing, China; Shandong University, CHINA

## Abstract

Based on a great number of literatures, we established the database about the Chinese endemic seed plants and analyzed the compositions, growth form, distribution and angiosperm original families of them within three big natural areas and seven natural regions. The results indicate that the above characters of Chinese endemic plants take on relative rule at the different geographical scales. Among the three big natural areas, Eastern Monsoon area has the highest endemic plants richness, whereas Northwest Dryness area is the lowest. For life forms, herbs dominate. In contrast, the proportion of herbs of Eastern Monsoon area is remarkable under other two areas. Correspondingly the proportions of trees and shrubs are substantially higher than other two. For angiosperm original families, the number is the highest in Eastern Monsoon area, and lowest in Northwest Dryness area. On the other hand, among the seven natural regions, the humid and subtropical zone in Central and Southern China has the highest endemic plants richness, whereas the humid, hemi-humid region and temperate zone in Northeast China has the lowest. For life forms, the proportion of herbs tends to decrease from humid, hemi-humid region and temperate zone in Northeast China to humid and tropical zone in Southern China. Comparably, trees, shrubs and vines or lianas increase with the same directions. This fully represents these characters of Chinese endemic plants vary with latitudinal gradients. Furthermore, as to the number of endemic plants belonging to angiosperm original families, the number is the most in humid and subtropical zone in Center and Southern China, and tropical zone in Southern China in the next place. In contrast, the endemic plant of these two regions relatively is richer than that of The Qinghai-Tibet alpine and cold region. All above results sufficiently reflect that the Chinese endemic plants mainly distribute in Eastern Monsoon area, especially humid and subtropical zone in Center and Southern China and tropical zone in Southern China. Furthermore, the flora of Eastern Monsoon area, in particular humid and subtropical zone in Center and Southern China and tropical zone in Southern China, is more ancient and original than that of Northwest Dryness area and Qinghai-Tibet alpine and cold area.

## Introduction

China has an land area of 9.60 million km^2^ spanning multiple climatic zones and a huge geographical area ranging from tropical to boreal zones and from very low altitudes to the world’s highest mountain [1]. Almost all of the different types of vegetation from rainforests to deserts are found in China [2,3]. This climatic and geographical diversity provides abundant habitats for plants and animals [4], and makes China become not only one of the richest countries in terms of plant biodiversity [5,6], but also a major center of survival speciation, and evolution for vascular plants in the world [3,7].

Formation and changes of land and sea, climate change, large-scale mountain or plateau uplift as well as regional ecological complications play different and important roles in speciation and diversification of flora. They all directly or indirectly affect speciation, differentiation, migration, extinction and preservation of flora. The early Paleozoic Caledonian movement makes land lift and Chinese continent disperse. Main mountains in northeast and northwest China and Qinling Mountains formed in later Paleozoic Hercynian tectonic period. In this period, Chinese continent was connected to the Siberian continental platform, and Tianshan Mountain was interlinked with mountains and plateau of central Asia. Tanggula mount, Karakoram and Hengduan mountains were formed during Yanshanian Tectonic Period between Mesozoic Jurassic and before Cenozoic [8]. In the Himalayan tectonic period, Himalayas and Taiwan mountains were present, and Qinghai-Tibet plateau was uplift. Meanwhile, ancient Mediterranean disappeared, and Eurasian continent was connected together as one. There was a collision and connection between India plate and Eurasia plate in this period. These strong tectonic constant activities of old Chinese continent casted modern Chinese complex topography and various environments, thus created high biodiversity [9]. China is the unique country that owns continuous various forest vegetation types with tropical, subtropical, temperate and boreal forest [10]. The continuity of forest vegetation provides advantageous conditions for biodiversity conservation [9].

In recently years, China has been developing in the field of biodiversity research including Species Catalogue, biodiversity monitoring, phylodiversity, biodiversity informatics and biodiversity conservation [11–15]. Endemism has become an increasingly common and irreplaceable surrogate for identifying the world’s biodiversity hotspots [16]. China is one of the countries with the highest number of threatened species in the world [17]. Of the World Wildlife Fund’s Global 200 most Critical and Endangered Ecoregions, 17 are located in or intersected with China [18]. Furthermore, of the 34 global biodiversity hotspots identified by Conservation International, four ones either intersect with or are located in China [19]. China flora is highly endemic [4,20]. Endemic species account for 52.1% of total seed plants species [21].

Many studies on biodiversity conservation and endemism of Chinese flora have carried out across China [21–27]. China seed flora has four endemic families [25,26], 243 endemic genera [27] and 15103 endemic species [28]. At species level, 52.1% seed plant species are endemic to China [21], and there are three centers of endemism in terms of endemic seed plants genera [27], eight hotspots of threatened plant species [23], 10 hotspot ecoregions [22], 11 terrestrial key areas for the biodiversity conservation [29], and 20 biodiversity hotspots for Chinese endemic woody seed plant species [24]. In those studies, all distribution patterns of natural species, endemic species or threatened species were analyzed based on administrative province or county units; all diversity hotspots or centers of species diversity were identified based on administrative or natural landform units. It is well known that China have a vast area and a variety of terrain, and thus the factors that affect the distribution of species diversity are various and complexed. Generally, in a large natural geographical area, the dominant factors affecting on distribution pattern of species diversity are relatively consistent. China has vast territory and obvious geographical area. The divisions of geographical areas are in accordance with water and heat energy. We would like to explore the relationship between water and heat energy and species diversity in the natural geographical area of China. However, we have no idea for diversity features and distribution patterns based on a natural geographic unit. Therefore, with a natural geographic unit as a basic spatial analysis unit, we analyzed distribution patterns diversity and differentiation degree of Chinese endemic seed plants across country.

## Materials and Methods

### Data set

According to checklist of Chinese endemic seed plant species [28], we compiled 18,157 endemic seed plants (including 14803 species, 288 subspecies and 3066 varieties) with distribution information and growth forms (S1 Appendix). Spatial units of distribution data of endemic plant contain province and county. Biological traits include growth forms of each plant. The main sources of distribution are as follows: a) *Flora of China* [30] and *Flora Reipublicae Popularis Sinicae* [31], b) local floras, plant checklists and relevant monographs, c) articles about plant taxonomy and distribution in journals and d) herbarium specimens. We inferred from 1044 flora, monographs, reports or theses, 516 articles, and 37 Herbaria by the end of 2012. All lists of reference and herbarium are provided in S2 Appendix.

**Fig 1:**
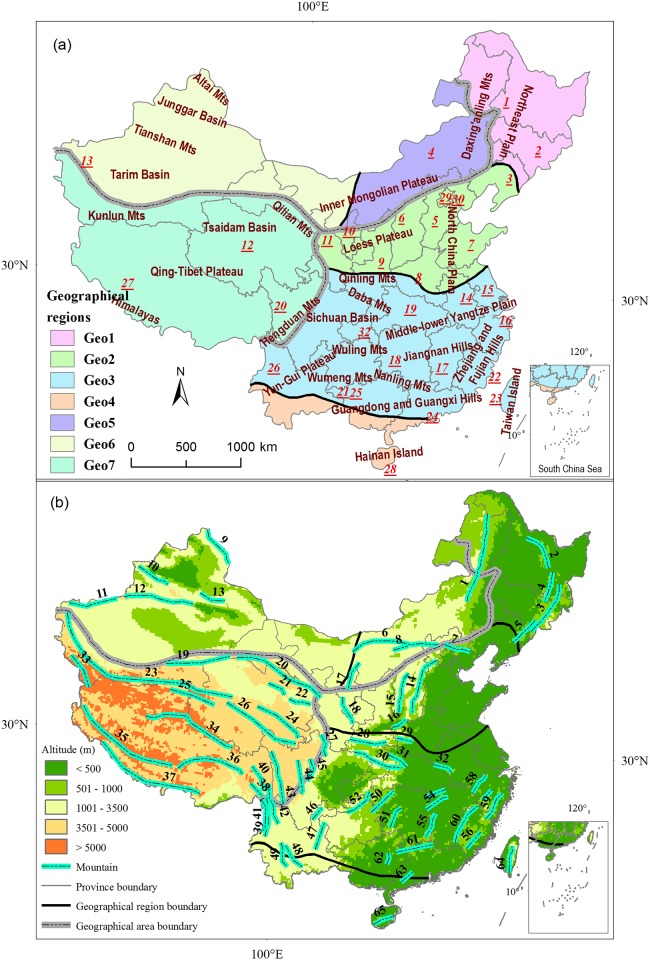
Maps of geography regionalization, administrative provinces, topography and major mountain ranges in China. (a) Locations of geography regionalization, administrative province and major morphostructures (Scarlet text). Abbreviation codes for geographical regions are in Table 1. Red number with underline identifies administrative province: 1 Heilongjiang, 2 Jilin, 3 Liaoning, 4 Inner Mongolia, 5 Hebei, 6 Shanxi, 7 Shandong, 8 Henan, 9 Shaanxi, 10 Ningxia, 11 Gansu, 12 Qinghai, 13 Xinjiang, 14 Anhui, 15 Jiangsu, 16 Zhejiang, 17 Jiangxi, 18 Hunan, 19 Hubei, 20 Sichuan, 21 Guizhou, 22 Fujian, 23 Taiwan, 24 Guangdong, 25 Guangxi, 26 Yunnan, 27 Tibet, 28 Hainan, 29 Beijing 30, Tianjin, 31 Shanghai, 32 Chongqing. (b) Elevation map of China and locations of main mountain ranges in China. According to Chinese physical geography [1], the topography is shown as five types, including plains (elevation < 500 m), low mountains (elevation of 500–1,000 m), middle mountains (elevation of 1,000–3,500 m), high mountains (elevation of 3,500–5,000 m) and very high mountains (elevation >5,000 m). Black number indicates main mountain ranges (Numbers are consistent with S1 Table). The inset in the right bottom of the figure shows the south boundary of China, includes all islands in the South China Sea. Albers projection.

According to map of geographical regions of China [32], we digitized the geographical regions of China using ArcGIS 9.3 [33]. The geographical regions of China were composed of three geographical areas, seven geographical regions (Table 1 and Fig 1). Thus, they amount to 7 spatial geographical units (Table 1 and Fig 1a). Based on distribution of endemic plants at both province and county units, we transformed distribution of species to a geographical unit. We carried out spatial analysis to overlap both maps of geographical regions and administrative provinces of China and to identify orientation relationship of geographical regions and province using Analysis Tools in ArcGIS. For provinces across more than one geographical region, we extracted centroids (latitude and longitude coordinates) of all counties within those provinces using Data Management Tools in ArcGIS. These centroids were joined into 7 geographical regions spatially with Analysis Tools, thus records of geographical regions for each species were obtained. Distribution information for the presence/absence of each species was documented for each geographical region. The data for the topography of China (Fig 1a) and main mountains [34] were derived from a digital elevation model (DEM) according to the criteria described in the Editorial Committee of China’s Physical Geography (1985).

**Table 1:** Seven geographical regions of China and their main climatic indexes.

Geographical area	Seven geographical region	Accumulated temperature ≥10°C*	Dryness degree*	Frost-free period (day)*
The Eastern Monsoon area (I)	The humid, hemi-humid region and temperate zone in Northeast China (Geo1)	1400–3200	0.5–1.2	<145
	The humid, hemi-humid region and warm temperate zone in Northern China (Geo2)	3200–4500	0.5–1.5	150–200
	The humid and subtropical zone in Center and Southern China (Geo3)	4500–7500	0.5–1.0	230–330
	The humid and tropical zone in Southern China (Geo4)	>7500	0.5–1.0	Whole year
The Northwest Dryness area (II)	The temperate grassland region in Inner Mongolia (Geo5)	2000–3000	1.2–4.0	<180
	The temperate and warm temperate desert in Northwest China (Geo6)	3200–4500	>4.0	<200
The Qinghai-Tibet alpine and cold area (III)	The Qinghai-Tibet alpine and cold region (Geo7)	<2000 vertical change	0.5–0.4 vertical change	<130

*Data source: The atlas of the physical geography of China [32].

### Data analysis

We analyzed the compositions of both the Chinese endemic plants and local endemic plants (the term “local endemic plants” indicates species that is only found in any single spatial geographical unit), and counted quantitative characteristics of each main group in terms of different growth forms. To detect the distribution pattern of Chinese endemic seed plant species in the entire country, we calculated Chinese endemic plants richness and local endemic plants richness separately. In addition, we compared and analyzed compositional characteristics of original taxa for each geographical region according to checklist of orders and families for original taxa [35]. All calculations and statistical charts were carried out in R 3.2.2 [36]. Distribution of geographical region was mapped using ArcGIS.

## Results

### Chinese endemic plants richness in natural geographical units

**Fig 2:**
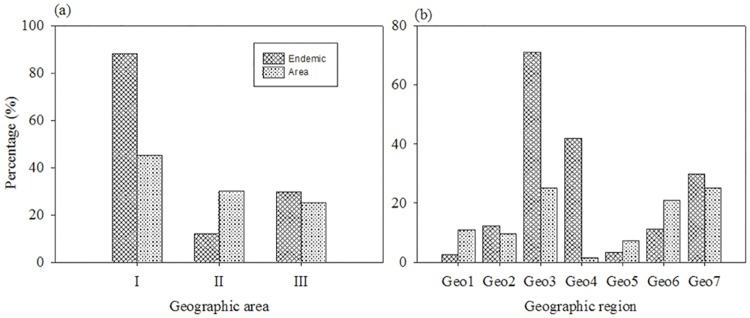
Comparison of percentage of endemics and area for 3 geographical area (a) and 7 geographical region (b), respectively. Abbreviation codes for geographical regions are in Table 1.

Chinese endemic seed plants are very unevenly distributed in natural geographical units. In three geographical areas, the Eastern Monsoon area is highest in number of endemic plants, while the Northwest Dryness area is lowest in that (Table 2). Ratio of number of endemic plants in the Eastern Monsoon area, the Qinghai-Tibet Alpine and Cold area, and the Northwest Dryness area 7.4:2.5:1. The Eastern Monsoon area accounted for 45.0% of the land, and distributed 88.0% of Chinese endemic plants; the Qinghai-Tibet Alpine and Cold area accounted for 25.0% of land, and distributed 29.8% of Chinese endemic plants; the Northwest Dryness area accounted for 30.0% of land, and contained less than 11.9% of Chinese endemic plants (Table 2, Fig 2a). Ratio of number of endemic plants per unit area in the Eastern Monsoon area, the Qinghai-Tibet Alpine and Cold area, and the Northwest Dryness area is 5:3:1. In seven geographical regions, Chinese endemic seed plants are especially unevenly distributed in four geographical regions across the Eastern Monsoon area; the humid and subtropical zone in Center and Southern China (Geo3) is the richest in endemic plants; the humid and tropical zone in Southern China (Geo4) is highest in endemic plants richness per unit area; the region Geo4 with 1.5% of the land contains about 41.9% of Chinese endemic seed plants (Table 3, Fig 2b).

**Table 2:** Number of Chinese endemic seed plants in three geographical area of China.

Geographical area	Number of plant endemic to China				Number of plant endemic to each area				Area (km^2^) *
	total	species	subspecies	variety	total	species	subspecies	variety	
The Eastern Monsoon area	15,986	1,3073	234	2,679	1,1956	9,790	145	2,021	4,320,000
The Northwest Dryness area	2,158	1,716	51	391	377	277	6	94	2,880,000
The Qinghai-Tibet alpine and cold area	5,418	4,439	124	855	1,770	1,436	47	288	2,400,000
					14,103	11,503	198	2,403	9,600,000

*Data source: China’s physical geography [1].

**Table 3:** Number of Chinese endemic seed plants in seven geographical regions of China.

Geographical region	Number of plant endemic to China				Number of plant endemic to each region				Area (km^2^)*
	total	species	subspecies	variety	total	species	subspecies	variety	
The humid, hemi-humid region and temperate zone in Northeast China	465	320	11	134	102	51	1	50	1,050,000
The humid, hemi-humid region and warm temperate zone in Northern China	2,210	1,718	51	441	282	173	7	102	920,000
The humid and subtropical zone in Center and Southern China	12,892	10,531	207	2,154	5,058	4,088	78	892	2,400,000
The humid and tropical zone in Southern China	7,601	6,404	83	1,114	2,273	1,985	14	274	140,000
The temperate grassland region in Inner Mongolia	573	425	13	135	95	57	1	37	680,000
The temperate and warm temperate desert in Northwest China	2,010	1,624	49	337	279	217	5	57	2,010,000
The Qinghai-Tibet alpine and cold region	5,418	4,439	124	855	1,771	1,436	47	288	2,400,000
Total					9,860	8,007	153	1,700	9,600,000

*Data source: China’s physical geography [1].

Chinese endemic plants are mostly limited to relatively narrow areas (Table 2). Only 7.4% of endemic plants are common species in three geographical areas, while 77.7% of endemic plants are only distributed in any single geographical area. At geographical region level, 54.3% of Chinese endemic plants (9860 taxa) are only present in any single geographical region. The temperate grassland region in Inner Mongolia (Geo5) is the lowest in local endemic plants richness; in contrast, the humid and subtropical zone in Center and Southern China (Geo3) is the highest (Table 3).

### Characteristics of growth forms of Chinese endemic plants in natural geographical units

**Fig 3:**
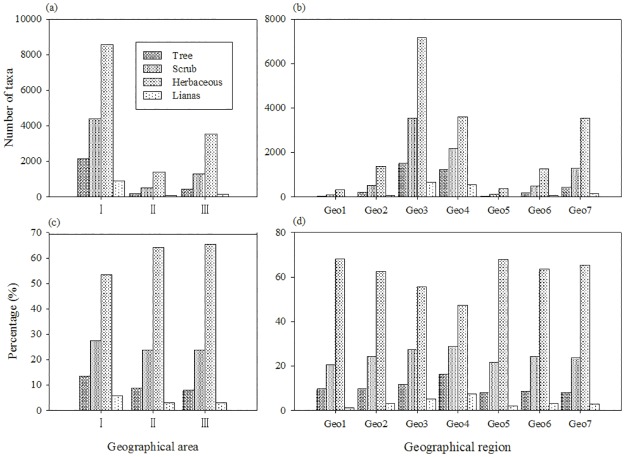
Comparison of the diversity of endemic taxa for different geographical units. (a) Number of endemic taxa in 3 geographical areas, (b) Number of endemic taxa in 7 geographical regions, (c) Percentage of endemic taxa in 3 geographical areas, (d) Percentage of endemic taxa in 7 geographical regions. Abbreviation codes for geographical regions are in Table 1.

Chinese endemic seed plants were divided into four kinds of growth forms: tree, shrub, herb, and liana or vine. Herb is dominant in any geographical region (Tables 4 and 5, Fig 3). Number of endemic herbaceous plants is the highest, and followed by shrub, tree, and liana or vine. The proportions of tree, shrub, and liana or vine in the Eastern Monsoon area are higher than that of in the Northwest Dryness area and in the Qinghai-Tibet alpine and cold area, and the proportion of herb, by contrast, obviously on the low side. In the Eastern Monsoon area, endemic plants richness of each growth form increases from the north to the south except for the humid and tropical zone in Southern China (Geo4). Number of endemic plant of the humid and subtropical zone in Center and Southern China (Geo3) is obviously much more than that of either the humid, hemi-humid region and temperate zone in Northeast China (Geo1) or the humid, hemi-humid region and warm temperate zone in Northern China (Geo2). The proportions of tree, shrub, and liana or vine increase from the north to the south in the Eastern Monsoon area, and the proportion of herb, by contrast, obviously decrease (Fig 3b and 3d).

**Table 4:** Number of Chinese endemic seed plants of the different life from and their percentages in 3 geographical areas.

Geographical area	Number of plant endemic to China				Percentage (%)			
	Tree	Shrub	Herbaceous	Lianas	Tree	Shrub	Herbaceous	Lianas
The Eastern Monsoon area	2,154	4,379	8,543	910	13.5	27.4	53.4	5.7
The Northwest Dryness area	188	515	1,386	69	8.7	23.9	64.2	3.2
The Qinghai-Tibet alpine and cold area	437	1,284	3,536	161	8.1	23.7	65.3	3.0

**Table 5:** Number of Chinese endemic seed plants of the different growth form and their percentages in 7 geographical regions.

Geographical region	Number of plant endemic to China				Percentage (%)			
	Tree	Shrub	Herbaceous	Lianas	Tree	Shrub	Herbaceous	Lianas
The humid, hemi-humid region and temperate zone in Northeast China	46	96	317	6	9.9	21	68	1.3
The humid, hemi-humid region and warm temperate zone in Northern China	218	538	1,383	70	9.9	24	63	3.2
The humid and subtropical zone in Center and Southern China	1,514	3,540	7,165	669	12	27	56	5.2
The humid and tropical zone in Southern China	1,244	2,188	3,605	564	16	29	47	7.4
The temperate grassland region in Inner Mongolia	47	125	389	12	8.2	22	68	2.1
The temperate and warm temperate desert in Northwest China	176	490	1,277	67	8.8	24	64	3.3
The Qinghai-Tibet alpine and cold region	437	1,284	3,536	161	8.1	24	65	3

### Distribution of original taxa of Chinese endemic plants in natural geographical units

There are 2120 Chinese endemic plants belonging to 19 original families [35] in China. These endemic plants account for 11.6% of total of Chinese endemic seed plants. These 19 original families (Table 6) are composed of two cosmopolitan geographical types, eight temperate geographical types and nine tropical geographical types. There are 153 endemic plants that are common in three geographical areas. These endemic plants are dominant in Ranunculaceae, Papaveraceae and Berberidaceae (Table 6). There are 1752 endemic plants (accounting for 82.6% of total endemic plants from original families) from original families in the Eastern Monsoon area. These endemic plants are mainly from Ranunculaceae, Lauraceae, Berberidaceae, Papaveraceae, Aristolochariaceae, Magnoliaceae, Menispermaceae and Annonaceae. There are 235 endemic plants (account for 11.1%) from 12 original families in the Northwest Dryness area. There are 720 endemic plants (accounting for 34.0%) from 13 original families in the Qinghai-Tibet alpine and cold area. Most of these endemic plants are belonged to Ranunculaceae, Papaveraceae, Berberidaceae and Lauraceae. Twenty four Chinese endemic plants main from Ranunculaceae, are present commonly in both the Eastern Monsoon area and the Northwest Dryness area, but are absent in the Qinghai-Tibet alpine and cold area; two hundred and fifty five Chinese endemic plants, which are main from Ranunculaceae, Berberidaceae, Lauraceae and Papaveraceae, are present commonly in both the Eastern Monsoon area and the Qinghai-Tibet alpine and cold area, but are absent in the Northwest Dryness area. Only two endemic plants, *Halerpestes tricuspis* var. *heterophylla* and *Berberis farreri*, are present commonly in both the Northwest Dryness area and the Qinghai-Tibet alpine and cold area, but are absent in the Eastern Monsoon area. One thousand three hundred and twenty Chinese endemic plants (accounting for 62.3%) from 18 original families are distributed restrictively within the Eastern Monsoon area. Only 56 Chinese endemic plants (accounting for less than 2.6%) form original families are limited in the Northwest Dryness area. There are 310 Chinese endemic plants (accounting for 14.6%) distributed limitedly within the Qinghai-Tibet alpine and cold area. These endemic plants mainly belong to Ranunculaceae, Papaveraceae, Berberidaceae and Lauraceae.

**Table 6:** Numbers of Chinese endemic seed plants from 19 original families in 7 geographical regions.

Code	Family	No. genus	No. endemic plants	Common endemic plants in three geographical areas	The Eastern Monsoon area				The Northwest Dryness area		The Qinghai-Tibet alpine and cold area
					The humid, hemi-humid region and temperate zone in Northeast China	The humid, hemi-humid region and warm temperate zone in Northern China	The humid and subtropical zone in Center and Southern China	The humid and tropical zone in Southern China	The temperate grassland region in Inner Mongolia	The temperate and warm temperate desert in Northwest China	The Qinghai-Tibet alpine and cold region
1	Annonaceae	12	39		0	0	9	36	0	0	2
2	Aristolochiaceae	4	71	2	1	4	61	36	0	2	13
3	Berberidaceae	7	279	21	1	28	219	80	2	25	113
4	Calycanthaceae	2	4		0	1	4	1	0	1	0
5	Chloranthaceae	1	14	3	0	3	14	7	0	3	6
6	Eucommiaceae	1	1		0	0	1	0	0	1	0
7	Hernandiaceae	1	11		0	0	8	8	0	0	0
8	Illiciaceae	1	19		0	1	15	11	0	1	3
9	Lardizabalaceae	5	26	1	0	1	20	13	0	3	8
10	Lauraceae	18	366	6	0	11	229	215	0	8	70
11	Magnoliaceae	6	59	3	0	0	47	31	0	3	6
12	Menispermaceae	5	48		0	1	25	33	0	0	5
13	Myristicaceae	1	1		0	0	0	1	0	0	0
14	Nymphaeaceae	1	1		0	1	1	0	0	0	0
15	Papaveraceae	6	277	33	5	41	164	54	6	35	158
16	Piperaceae	2	41		0	0	22	29	0	0	4
17	Ranunculaceae	29	839	84	27	129	548	182	37	135	329
18	Saururaceae	2	2		0	0	2	0	0	0	0
19	Schisandraceae	2	22		0	2	20	7	0	2	3
	Total	106	2120	153							

Only *Ranunculus furcatifidus* and *Clematis akebioides*, which both belong to Ranunculaceae family, are common endemic plants in seven geographical regions. Four Chinese endemic plants, which all belong to Ranunculaceae family, are common in six geographical regions. Thirty Chinese endemic plants, which are dominant in Ranuculaceae, are common in five geographical regions. Eighty six Chinese endemic plants are common in four geographical regions. These endemic plants belong to Ranunculaceae, Papaveraceae, Berberidaceae. There are 1409 endemic plants (accounting for 66.5%) from original families present in the humid and subtropical zone in Center and Southern China (Geo3). Among of them, 653 plants (accounting for 30.8%) are restricted in this geographical region. There are 744 endemic plants (accounting for 35.1%) from original families present in the humid and tropical zone in Southern China (Geo4). Among of them, 255 plants (accounting for 12.0%) are restricted in this geographical region.

## Discussion

Chinese endemic seed plant species are very unevenly distributed in three geographical areas and seven geographical regions. The Northwest Dryness area is arid because Qinghai-Tibet Plateau, Qinling Mountains, Taihang Mountain, and Yanshan and Da Hinggan Ling Mountain (Fig 1) prevent the monsoon winds from passing south and east China to north and west China, thus maintaining a dried climate and deficient moisture in summer [1]. Although Arctic Ocean and Siberian cold airs could reach this area in winter, there is still deficient in moisture due to the Arctic Ocean far away. Therefore, grassland and desert are its dominant vegetation, and most of these endemic plants are drought-tolerant in this area. The Qinghai-Tibet alpine and cold area is arid and cold because the humid monsoon winds from south and east China (e.g. the Pacific warm current) can’t reach due to the average altitude with more than 4000 m of this plateau is too high [1]. Therefore, most of these endemic plants are cold resistant in this area. In contrast, the Eastern Monsoon area, especially in the humid and subtropical zone in Center and Southern China region (Geo3 in Fig 1a) and the humid and tropical zone in Southern China (Geo4 in Fig 1a), is humid, and usually invaded by ocean air. Qinling Mountains and Hengduan Mountains prevent the monsoon winds from south and east China to north and west China, thus maintaining a wet climate and adequate moisture in summer. These mountains also prevent Siberian cold air masses from reaching south China, thus maintaining the warm climate in winter [37]. Moreover, both regions are absence of severe continental glaciations during the Plio-Pleistocene periods [38]. These favorable conditions maintain greater variety of habitats with in the both regions and thus probably accelerate the speciation, differentiation and preservation for the species living in these regions because of high habitat heterogeneities [38,39]. Therefore, Chinese endemic seed species are mainly contracted in this area. Most of endemism centers and diversity hotspots of Chinese endemic flora [40] are located in the both regions. Consequently, the main reason that drove spatial distribution patterns of Chinese endemic flora is that natural physical terrains (plateaus and mountains) change the movement of atmosphere and cause changes in precipitation and temperature. Furthermore, this probably shows that drought and coldness are not convenient to preservation and differentiation of Chinese endemic seed flora.

The proportions of tree, shrub, and liana or vine in the Eastern Monsoon area are higher than that of in the Northwest Dryness area and in the Qinghai-Tibet alpine and cold area. On the contrary, the proportion of herb in the Eastern Monsoon area is less than that of in the Northwest Dryness area and in the Qinghai-Tibet alpine and cold area. Herb is considered to be more evolutionary group than tree [41]. On this basic, we think, Chinese endemic seed flora is relative old in the Eastern Monsoon area, but relatively young in the Northwest Dryness area and the Qinghai-Tibet alpine and cold area. This difference in development of endemic flora probably results from topography, neotectonic movements and glaciation [27]. The Eastern Monsoon area had not obvious uplift in nontectonic movement, and did not suffer the Quaternary glaciation [38]. Therefore, flora suffered disasters are relatively small, and endemic flora preserve relatively well. On the contrary, the Northwest Dryness area and the Qinghai-Tibet alpine and cold area had obvious uplift in nontectonic movement, especially in late Pliocene and early Pleistocene, and change original landforms completely. Moreover, modern glacier and the Quaternary glacier distribute widely in these both areas [1]. These glaciers force original plants to migrate or extinguish. Meanwhile, the birth of new environment prompted speciation and differentiation of original groups, thus endemic flora are relatively young in these areas [27].

Both Chinese endemic plants richness and local endemic plants richness are higher in the humid and subtropical zone in Center and Southern China than that in the Qinghai-Tibet alpine and cold area. This might implicate that endemic flora in the humid and subtropical zone in Center and Southern China is older than that in the Qinghai-Tibet alpine and cold area [27]. Considering Chinese endemic plants richness per unit area, the humid and tropical zone in Southern China region (Geo4) is higher than the humid and subtropical zone in Center and Southern China (Geo3) and the Qinghai-Tibet alpine and cold area. Endemic flora in the humid and tropical zone in Southern China region (Geo4) has more tropical geographical types. Therefore, we are convinced that endemic flora of this region is enough to reflect tropical and the historical development of antiquity [27].

## Conclusion

Chinese endemic plants take on relative rule at the different geographical scales. The Chinese endemic plants mainly distribute in Eastern Monsoon area, especially humid and subtropical zone in Center and Southern China and tropical zone in Southern China. The flora of Eastern Monsoon area, in particular humid and subtropical zone in Center and Southern China and tropical zone in Southern China, is more ancient and original than that of Northwest Dryness area and Qinghai-Tibet alpine and cold area.

## Supporting Information

S1 Table List of China’s major mountain ranges. (DOC)

S1 Appendix Growth forms and distributions of 18157 Chinese endemic seed plants. (XLSX)

S2 Appendix Reference and herbarium lists that we have consulted to establish the Chinese seed plant species inventory and to collect their distribution information. (DOC)
