# Supplementary material for: Species Diversity Distribution Patterns of Chinese Endemic Seed Plants Based on Geographical Regions
Source: PLoS One. 2017 Jan 23;12(1):e0170276. doi: 10.1371/journal.pone.0170276 (PMC5256866; doi:10.1371/journal.pone.0170276)
Supplement: S1 Table — (DOC) [file pone.0170276.s001.doc]

**Table S1** List of China’s major mountain ranges (Wang *et al*., 2004)

| No. | Name | No. | Name |
| --- | --- | --- | --- |
| 1 | Daxing'anling Mountains | 34 | Danggula Mountains |
| 2 | Xiaoxing'anling Mountains | 35 | Gangdisi Mountains |
| 3 | Changbai Mountains | 36 | Nyainqntanglha Mountains |
| 4 | Zhangguangcailing Mountains | 37 | Himalayas |
| 5 | Longgang Mountains | 38 | Boshula Mountains |
| 6 | Yinshan Mountains | 39 | Gaoligong Mountains |
| 7 | Yanshan Mountains | 40 | Taniantaweng Mountains |
| 8 | Daqing Mountains | 41 | Nushan Mountains |
| 9 | Altai Mountains | 42 | Yunling Mountains |
| 10 | Poluokenu Mountains | 43 | Shaluli Mountains |
| 11 | South branch of Tianshan Mountains | 44 | Daxue Mountains |
| 12 | Tianshan Mountains | 45 | Qionglai Mountains |
| 13 | Bogeda Mountains | 46 | Wulian Mountains |
| 14 | Taihang Mountains | 47 | Wumeng Mountains |
| 15 | Luliang Mountains | 48 | Ailao Mountains |
| 16 | Zhongtiao Mountains | 49 | Wuliang Mountains |
| 17 | Helan Mountains | 50 | Wuling Mountains |
| 18 | Liupan Mountains | 51 | Xuefeng Mountains |
| 19 | Aerjin Mountains | 52 | Dalou Mountains |
| 20 | Qilian Mountains | 53 | Mufu Mountains |
| 21 | Qinghainan Mountains | 54 | Jiuling Mountains |
| 22 | Laji Mountains | 55 | Luoxiao Mountains |
| 23 | Kunlun Mountains | 56 | Daiyun Mountains |
| 24 | Animaqin Mountains | 57 | Xianxialing Mountains |
| 25 | Kekexili Mountains | 58 | Tianmu Mountains |
| 26 | Bayankala Mountains | 59 | Yandang Mountains |
| 27 | Minshan Mountains | 60 | Wuyi Mountains |
| 28 | Qinling Mountains | 61 | Nanling Mountains |
| 29 | Funiu Mountians | 62 | Dayao Mountains |
| 30 | Daba Mountains | 63 | Yunwu Mountains |
| 31 | Wudang Mountains | 64 | Yushan Mountains |
| 32 | Dabie Mountains | 65 | Wuzhi Mountains |
| 33 | Karakorum Mountains |  |  |
